# Promoting and supporting breastfeeding in a protracted emergency setting—Caregivers' and health workers' perceptions from North-East Nigeria

**DOI:** 10.3389/fpubh.2023.1077068

**Published:** 2023-06-02

**Authors:** Nieves Amat Camacho, Abdullahi Chara, Emily Briskin, Umberto Pellecchia, Htet Aung Kyi, Maria Livia de Rubeis, Faisal Hussain, Tasneem Ahmed, Oluwakemi F. Ogundipe, Chiara Burzio, Uba Kamis, Lawan M. Bukar, Johan Von Schreeb, Ourania Kolokotroni, Francesco Della Corte, Temmy Sunyoto

**Affiliations:** ^1^Center for Research in Healthcare in Disasters, Global Public Health Department, Karolinska Institutet, Stockholm, Sweden; ^2^Center for Research and Training in Disaster Medicine, Humanitarian Aid, and Global Health, Università del Piemonte Orientale, Novara, Italy; ^3^Médecins Sans Frontières, Operational Center Brussels, Abuja, Nigeria; ^4^Luxembourg Operational Research Unit, Médecins Sans Frontières, Operational Center Brussels, Luxembourg, Luxembourg; ^5^Médecins Sans Frontières, Operational Center Brussels, Brussels, Belgium; ^6^Nutrition Unit EPID, Borno State Primary Health Care Development Agency, Maiduguri, Nigeria; ^7^Faculty of Clinical Sciences, University of Maiduguri, Maiduguri, Nigeria; ^8^Department of Nursing, School of Health Sciences, Cyprus University of Technology, Limassol, Cyprus

**Keywords:** breastfeeding support, infant malnutrition, humanitarian settings, breastfeeding, North-East Nigeria

## Abstract

**Background:**

Breastfeeding (BF) should be protected, promoted, and supported for all infants in humanitarian settings. The re-establishment of exclusive BF is also a central part of the management of acutely malnourished infants under 6 months (<6 m). Médecins Sans Frontières (MSF) runs a nutrition project in Maiduguri, a protracted emergency setting in North-East Nigeria. This study aimed to explore caregivers' (CGs) and health workers' (HWs) perceptions of BF practice, promotion, and support among CGs with infants <6 m in this setting.

**Methods:**

We conducted a qualitative study using in-depth interviews and focus group discussions combined with non-participant observations. Participants included CGs of young infants enrolled in MSF nutritional programs or who attended health promotion activities in a displacement camp. MSF HWs were involved at different levels in BF promotion and support. Data were collected involving a local translator and analyzed using reflexive thematic analysis directly from audio recordings.

**Results:**

Participants described how feeding practices are shaped by family, community, and traditional beliefs. The perception of breastmilk insufficiency was common and led to early supplementary feeding with inexpensive but unsuitable products. Participants often linked insufficient breastmilk production with poor maternal nutrition and stress, in a context shaped by conflict and food insecurity. BF promotion was generally well received but could be improved if tailored to address specific barriers to exclusive BF. Interviewed CGs positively valued BF support received as part of the comprehensive treatment for infant malnutrition. One of the main challenges identified was the length of stay at the facility. Some participants perceived that improvements in BF were at risk of being lost after discharge if CGs lacked an enabling environment for BF.

**Conclusion:**

This study corroborates the strong influence of household and contextual factors on the practice, promotion, and support of BF. Despite identified challenges, the provision of BF support contributes to improvements in BF practice and was positively perceived by CGs in the studied setting. Greater attention should be directed toward providing support and follow-up for infants <6 m and their CGs in the community.

## 1. Introduction

Infants aged <6 months of age (<6 m), with unique food and fluid requirements and an immature immune system, are increasingly vulnerable during humanitarian emergencies (1, 2). Non-breastfed and partially breastfed infants are at higher risk of morbidity and mortality, especially where access to safe breastmilk substitutes, potable water, hygiene, and healthcare services is limited (3–5).

The Operational Guidance for Infant and Young Child Feeding in Emergencies recommends the early initiation of breastfeeding (BF) in all newborns, the transition from mixed to exclusive BF, and the continuation of exclusive BF until 6 months of age. For non-breastfed infants, the guidelines advise considering alternative options such as relactation, wet nursing, or the use of donor human milk. In cases where mothers cannot breastfeed temporarily or definitively, the decision to provide infant formula should be individually assessed and include a package of support for artificial feeding (6).

The re-establishment of exclusive BF is also considered a central part of the management of malnourished infants <6 m, although current recommendations are based on limited and low-quality evidence (7). Increased knowledge of prevention and treatment options is needed, including effective ways to support the re-establishment of BF (8–11).

Considerable barriers can hinder the uptake of BF during humanitarian emergencies. They include the deterioration of maternal wellbeing (due to displacement, stress, and gender-based violence), a lack of privacy or access to lactation support, and the inappropriate distribution of infant formula (8, 12–14). In addition, supporting BF can be challenging for health professionals regardless of their specialization, as they face a demand that requires knowledge, practical skills, and cultural sensitivity, for which they may not feel properly prepared (15).

Unfortunately, scarce literature informs the feasibility, acceptability, and effectiveness of different strategies to promote and support BF during humanitarian emergencies (13, 16, 17). The impact of BF counseling on stressed, traumatized, or malnourished mothers and infants also remains unstudied (18).

For more than a decade, the population in North-East Nigeria has been affected by an ongoing humanitarian emergency driven by a conflict involving Nigerian government forces and armed opposition groups. Nearly 2.2 million people were internally displaced there in mid-2021, a region also exposed to drought, flooding, and disease outbreaks. The loss of livelihoods and food security was further exacerbated by the COVID-19 pandemic (19). Between January and December 2022, over 1.3 million children under 5 years were expected to suffer from acute malnutrition in North-East Nigeria (including over 300,000 cases of severe malnutrition) and more than 152,000 pregnant and lactating women could be acutely malnourished and needing nutrition support (20). The international medical humanitarian organization, Médecins Sans Frontières (MSF), runs a project in Maiduguri, North-East Nigeria, providing nutrition and health support to displaced and host populations in the area.

This study aimed to explore caregivers' (CGs) and health workers' (HWs) experiences and perceptions of BF practice, promotion, and support among infants <6 m in North-East Nigeria. The results can contribute to a strategy for BF promotion and support among CGs with healthy and malnourished infants <6 m in this context.

## 2. Materials and methods

This qualitative study followed the approach of reflexive thematic analysis proposed by Braun and Clarke, situated within an interpretive qualitative paradigm. This method, increasingly used for applied qualitative health research, respects and expresses the subjectivity of participants' accounts, while acknowledging the reflexive influence of researchers' interpretations (21–23). We adopted a critical orientation to examine how the wider social context may have shaped people's constructed meanings (24). This article adheres to the consolidated criteria for reporting qualitative research (COREQ) checklist (25).

### 2.1. Study setting

Borno is one of the states in North-East Nigeria, predominantly inhabited by Kanuri, Hausa, Fulani, Babur, Shuwa, and Marghi ethnic groups. Most of the population are Muslim, with smaller Christian and traditionalist minorities. Households are commonly headed by men, and polygyny is estimated to be present in approximately 23% of households (26).

This study took place in an MSF project run in Maiduguri, the capital of Borno State. The project focuses on nutrition and health support for internally displaced people (IDP), mainly providing the following services:

- Ambulatory therapeutic feeding center (ATFC): the center has the capacity for 150 consultations/day, managing severely malnourished children without medical complications and following up with malnourished infants <6 m after discharge.
- Inpatient therapeutic feeding center (ITFC): the center has a 120-bed capacity, including a dedicated ward for infants <6 m (approximately 15 beds).
- Outreach activities covered 10 informal IDP camps providing ATFC, outpatient consultations, sexual and reproductive health, water and sanitation, health promotion, mental healthcare, disease surveillance, and a referral system.

BF promotion targets CGs of both malnourished and non-malnourished infants. It takes place in the community (during outreach health promotion activities) and at the hospital (in waiting areas and hospitalization wards), as part of the general health promotion strategy including other topics. Community health educators and health promotors undertake BF promotion through individual or group talks using pre-determined messaging, flipcharts, or educational videos. During admission to the ITFC ward for malnourished infants <6 m, comprehensive BF support is provided to mothers or wet nurses aiming to re-establish exclusive BF. Discharge from the ITFC depends, in part, on achieving and maintaining weight gain on exclusive BF. Specific support includes frequent encouragement of BF, assistance on BF technique (correct positioning and latching), and breastmilk stimulation through supplementary suckling technique while ensuring appropriate maternal nutrition and rest. Mental health support and psycho-stimulation are provided by mental health counselors in the ward as well as in private dedicated spaces when needed. A team of three health workers is dedicated to this ward in each shift, normally comprising one nurse, one midwife, and one nutritional assistant. Doctors perform daily ward rounds and follow up with infants when necessary.

### 2.2. Participant selection and recruitment

We purposively selected CGs of infants <6 m (mothers, wet nurses, and close family members) attending the MSF hospital for ATFC follow-up visits or admitted at the ITFC, and during health promotion activities at Mashidimami II, an informal IDP camp. We also selected HWs employed at the MSF project, directly involved in BF promotion or support for CGs with infants <6 m or supervising those activities. Study participants were selected aiming to include different socio-demographic characteristics and job profiles and according to logistical feasibility (availability to participate in the study during the data collection period).

**Figure 1:**
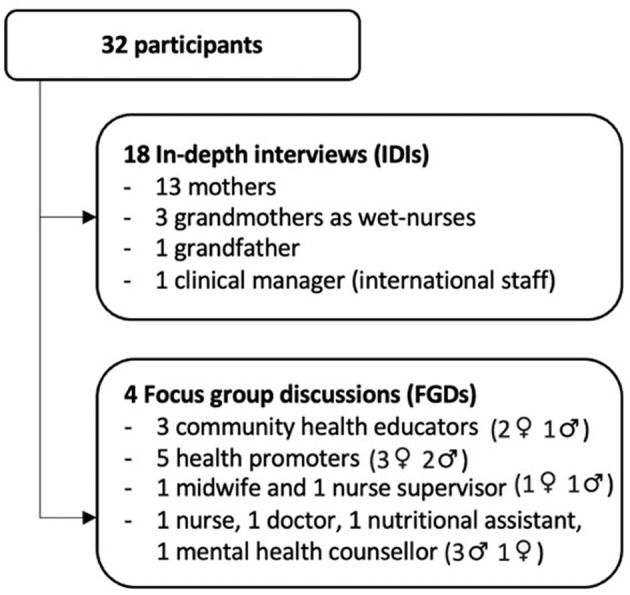
Study participants and data collection methods.

Eligible participants were approached in person by the first author (NAC), and a translator when needed, to explain the study purpose and data collection process and to ask if they would like to participate voluntarily. All invited participants agreed to take part in the study (Figure 1). The recruitment of participants continued until the saturation of salient concepts was observed (27).

### 2.3. Data collection

Data were collected in March 2022 by NAC with the support of a local translator and a young female community health educator. She was trained for the study and was involved in the preparation of in-depth interviews (IDIs) and revision of audio recordings, to check the consistency of translation and discuss methodological reflexivity within the process (28). A sample of her translations was also assessed by another research team member to ensure translation accuracy.

To favor a confidential environment for more in-depth answers, we choose to conduct IDIs with CGs. Before starting IDIs, basic socio-demographic information was collected from participants and documented on a written form. We used an interview guide with questions and probes (Supplementary material 1), which was tested on two participants; some questions were added as a result. The guide contained questions about infant feeding practices, barriers and enablers to BF, experiences receiving BF promotion and support, and preferences/suggestions for feeding support. The IDIs lasted from 20 to 45 min, were conducted in either Hausa or Kanuri languages, and took place at a private area within the MSF hospital and the informal IDP camp.

Four focus group discussions (FGDs) were organized to explore HWs' experiences and perspectives. Discussions were in English, led by NAC at the MSF hospital, and took from 45 to 80 min. The FGDs were guided by a script containing five key questions with probes comprising: perceptions about common feeding practices; perceived barriers to BF; experiences promoting and supporting BF; barriers to BF promotion and support; perceptions on how BF promotion and support could be improved (Supplementary material 1). An IDI was conducted with one clinical manager to get more in-depth information. The data collection process is summarized in Figure 1. All IDIs and FGDs were audio-recorded with prior authorization from interviewees.

During the data collection period, NAC was immersed in project activities, especially in the ITFC ward dedicated to infants <6 m. She recorded her observations of contextual and personal dynamics in field notes according to recommendations by Phillippi and Lauderdale (29). Notes of non-verbal information and impressions during IDIs and FGDs were not taken during the interviews but were written afterward. At the end of each day, NAC listened to audio-recording samples and revised and expanded the field notes with more thoughtful perceptions (reflective journaling). Most prominent answers were discussed with other field workers involved in the study to gather their views and adjust the data collection process if needed (adding new probes to the IDI guide or FGD script).

### 2.4. Data analysis

We performed direct analysis from the audio recordings, which included original data in English and the recorded translations from Hausa/Kanuri to English. Direct coding from audio files can provide a high level of detail and the ability to capture intonations while reducing coding time (30, 31). Rapid qualitative techniques have been found suitable for complex humanitarian settings to enable the timely acquisition of data during operations (32, 33).

As shown in Table 1, reflexive thematic analysis followed the recommended steps (21, 34). Data were open-coded by NAC, mostly inductively, to best represent its meaning as communicated by the participants. A deductive approach ensured that themes were relevant to answer research questions and objectives (21). Semantic (more explicit) and latent (underlying) meanings were considered, and sometimes extracts were coded twice. Microsoft OneNote was used to code the data from audio files and combine it with field notes (35).

**Table 1:** Phases of reflexive thematic analysis [adapted from Braun and Clarke (34)].

**Phases**	**Description of the process**
1. Familiarization with the data	Listening and re-listening to audio recordings and noting down initial ideas
2. Generating codes	Open coding relevant features of the data on a systematic fashion across the entire dataset
3. Searching for themes	Collating codes into potential themes and subthemes
4. Reviewing themes	Checking if the themes work in relation to the coded extracts and the entire data set
5. Defining and naming themes	Refine the specificities of each theme, and the overall story the analysis tells
6. Producing the report	Selecting and transcribing meaningful examples from the data, final analysis of selected extracts, relating the analysis to the research question

Audio files, field notes, and the coding matrix were shared among a subgroup of researchers, who helped to refine the themes and subthemes. The results were discussed in a consultative meeting that included the study team members and MSF staff at different levels.

We strived for the trustworthiness of findings by following several steps throughout all phases of reflexive thematic analysis (thick descriptions, prolonged data engagement, reflective journaling, and peer debriefing) as suggested by Nowell et al. (36).

### 2.5. Research team reflexivity

NAC is a female nurse and PhD student from Europe, with some previous experience in qualitative research and humanitarian fieldwork. She is also a mother. Both NAC and the translator were female, which favored trust building with female participants during data collection. The study was conditioned by being conducted within the frame of a project run by an international NGO providing free healthcare. Socio-cultural differences between the principal investigator and the study participants were noteworthy but partly balanced by the presence and inputs of the translator and the inclusion of local colleagues within the research team who could better grasp contextual nuances.

### 2.6. Ethics considerations

Ethics approval was granted by the MSF Ethics Review Board (ID 2207) and the Borno State Health Research Ethics Committee, Nigeria (Approval No. 113/2022). All study participants granted their written, informed consent before data collection. For illiterate participants, we read the consent form in Hausa or Kanuri languages, and consent was recorded using a thumbprint, witnessed by the translator. We offered in-kind compensation for CGs after they participated in the study. The audio recordings and the signed informed consent forms are stored in a password-protected folder on an MSF server, where they will be kept for 10 years before being deleted.

## 3. Results

The characteristics of CGs participating in the study are summarized in Table 2. Most mothers were grand multipara, with a median age of 30 years (IQR = 26–39) and seven children (IQR = 4–7). Only four mothers declared to be exclusively BF at the time of the interview, but two of them reported BF difficulties. Regarding HWs, half of the participants were female, aged between 20 and 40 years, and most had worked on the project for more than 2 years.

**Table 2:** Characteristics of caregivers participating in the study.

**Participant**	Age^*^	**IDP status**	**Education level**	No. children^*^	**Interview setting**	Days ITFC^**^	**Infant feeding practice before reaching MSF services**
Mother 1	30–35	Non-IDP	Illiterate	5–7	ITFC	6	EBF (inadequate)
Mother 2	30–35	Non-IDP	Basic education	8–10	ITFC	19	BF + powdered milk (cup)
Mother 3	25–30	IDP	Illiterate	4–6	ITFC	4	BF + formula with cereals (cup+spoon)
Mother 4	35–40	Non-IDP	Illiterate	8–10	ITFC	17	BF + powdered milk (cup)
Mother 5	35–40	Non-IDP	Illiterate	5–7	ITFC	6	BF + powdered milk (cup)
Mother 6	25–30	Non-IDP	Basic education	2–4	ITFC	19	Powdered milk (cup)
Grandmother 1 (wet-nurse)	40–45	Non-IDP	Illiterate	5–7	ITFC	4	BF + fresh cow milk (bottle)
Mother 7	30–35	Non-IDP	Illiterate	5–7	ITFC	6	BF + infant formula (bottle)
Mother 8	35–40	IDP	Illiterate	8–10	ATFC		BF + powdered milk
Mother 9	20–25	Non-IDP	Basic education	2–4	ATFC		BF + infant formula
Grandmother 2 (wet-nurse)	45–50	IDP	Illiterate	8–10	ATFC		BF + powdered milk + Porridge
Grandmother 3 (wet-nurse)	45–50	IDP	Illiterate	8–10	ATFC		BF + powdered milk + Porridge
Mother 10	35–40	IDP	Illiterate	5–7	IDP camp		EBF (inadequate)
Mother 11	20–25	IDP	Illiterate	2–4	IDP camp		EBF
Mother 12	35–40	IDP	Illiterate	5–7	IDP camp		BF + water
Mother 13	30–35	IDP	Illiterate	5–7	IDP camp		EBF
Grandfather	50–55	IDP	Primary level	5–7	IDP camp		-

BF, breastfeeding; EBF, exclusive breastfeeding; ITFC, Inpatient Therapeutic Feeding Center; IDP, internally displaced people.

* Intervals are shown for age and number of children.

** Number of days admitted at ITFC when interviewed.

**Figure 2:**
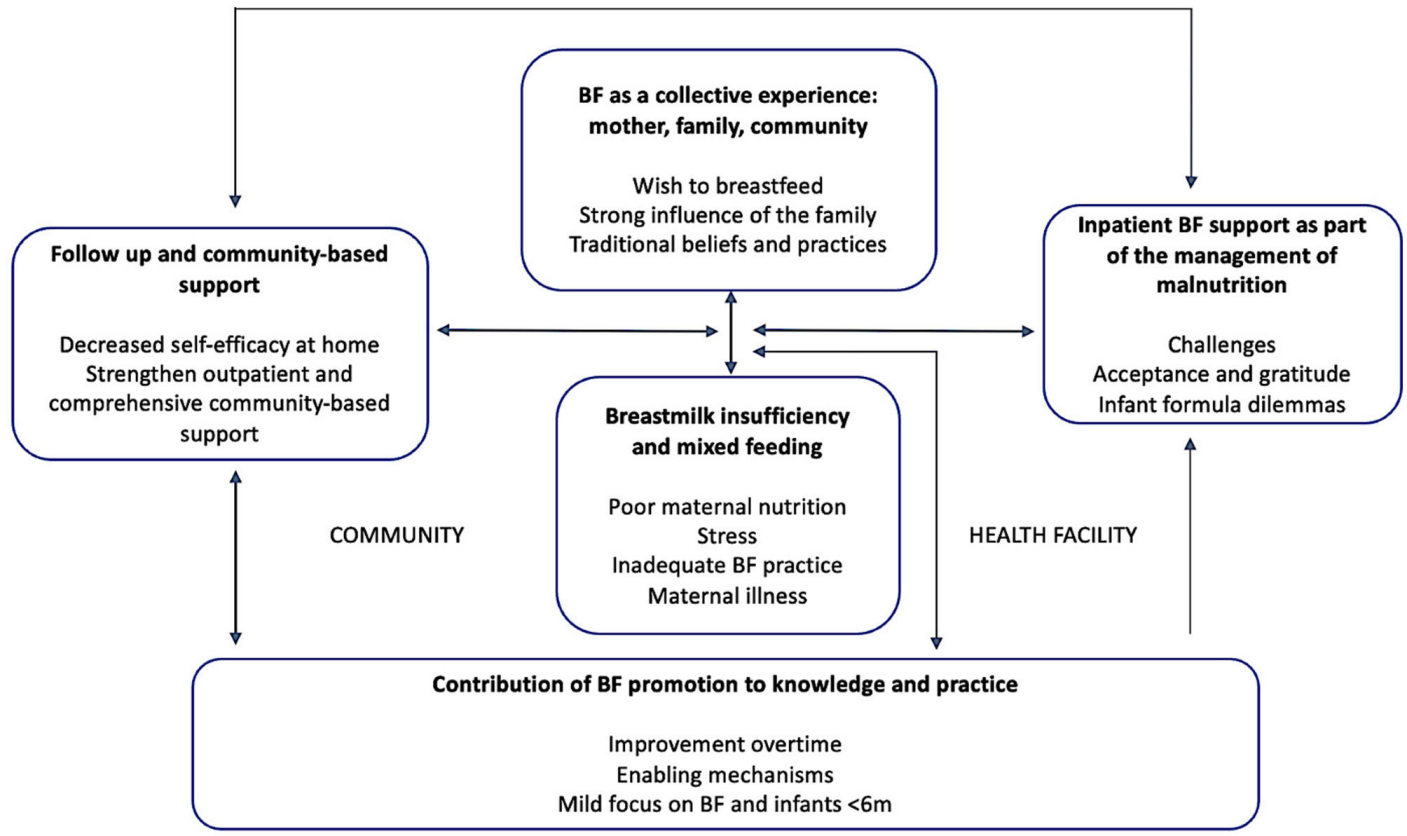
Thematic map including themes and subthemes.

The results were grouped into five overarching themes with subthemes (Figure 2) and are described below with illustrative quotes from study participants. To ensure the confidentiality and anonymity of the HWs, quotes were identified as coming from health promotors (referring to health promotors, community health educators, or mental health counselors) or clinical staff (referring to doctors, nurses, midwives, or nutritional assistants).

### 3.1. BF collective experience: mother, family, and community

Overall, there was a positive perception of BF; it was seen as a natural part of motherhood and women felt as if it was a duty. Mothers expressed their wish to breastfeed their babies even if they encountered difficulties. On the other hand, HWs identified a small percentage of mothers who preferred not to breastfeed for different reasons, such as the impact of BF on body image or the assumed superiority of other animal milk (cow or goat), infant formula, or therapeutic milk.

“*I feel happy whenever I breastfeed the baby, and breastfeeding is very good… I don't see the reason why I would give birth and I cannot breastfeed the baby.” Mother3*

“*Some women do not want to breastfeed because [they say]: we feel that when we breastfeed our breasts fall down, so it's no more attractive to the husband.” Clinical staff*

In contrast to the mothers' wishes, HWs perceived infant feeding practices, and health-seeking behaviors were frequently determined by the elder members of the family or by the husband. When a mother's and family's preferences contradicted each other, the preferences of the family were usually followed. In some cases, the family enabled and encouraged exclusive BF. The main support for BF expected from husbands was the provision of extra food for mothers while lactating. From the HW's perspective, marital dynamics such as polygyny, early marriage, or domestic violence also shaped mothers' ability and desire to breastfeed.

“*The tradition here is that you have to go with what the husband wants or what the mother-in-law wants. So even if you as a mother want to do exclusive breastfeeding, if the husband say no or his mother say no, you cannot do it.” Health promotor* [sic]

Infant feeding practices seemed to be greatly influenced by specific **traditions and beliefs**, which differed among different communities and ethnic groups. Several traditional practices contributed to the delayed start of BF. Traditional medicine and traditional healers were well-rooted inside communities, and they were the first choice for most participants with health concerns including BF. If there was no improvement following traditional healers' advice or the health issues were perceived to be more serious, people sought care in hospitals or formal healthcare facilities. Sometimes traditional healers discouraged exclusive BF or advised them to stop BF.

“*In our tradition, if a baby is born, she will not be breastfed till after 3 days. On the fourth day, they will start breastfeeding the baby. (…) we give cow milk, fresh milk, and water during these 3 days.” Grandmother 1*

“*I had enough breastmilk but when I went to traditional healers, they told me that I had enough breast, but the breast is not that nutritious that can be sufficient for the baby.” Mother 3*

Two grandmothers explained how they tried to breastfeed a grandchild for the first days of life, while the baby's mother was sick post-partum. In both cases, the mother died, and grandmothers became the main CGs and wet nurses who could partially breastfeed. However, wet nursing appears to be accepted only when a mother has died and when the wet nurse is the grandmother or some other close relative (a sister or a sister-in-law). In other cases, wet nursing was rarely considered.

“*In my culture is not accepted, for me to give the baby to nursing mother to breastfeed.” Mother 9 (mother with twins on formula feeding)* [sic]

“*They will not agree [a family member to wet-nurse her baby]. My sisters have their own children so I don't think they will agree to breastfeed my baby.” Mother5*

### 3.2. Breastmilk insufficiency and mixed feeding

Even when mothers initially wished to breastfeed, they commonly felt they could not produce enough breastmilk to feed their children appropriately. This perception of breastmilk insufficiency was driven by cues like babies frequently crying, not falling asleep after BF, or not gaining weight as expected. As a solution, mothers' families provided unsuitable products for infant feeding that were available and affordable to them. Most families could not afford infant formula, so they often used sachets of powdered milk (“Cowbell” commercial name), or a porridge called “pap”.

“*The breastmilk is not enough for the baby, because he is crying all the time, day and night, and I do not have money to buy infant formula, so I decided to buy Cowbell milk, which is the one that adults use.” Mother4*

There was also a belief that breastmilk was not sufficient for the baby and that other supplements must be added, especially water, due to the extreme weather conditions (very hot and dry). In this setting of food insecurity, all CGs and HWs strongly believed that maternal nutrition critically influenced breastmilk production and its quality. This was the most commonly described issue among CGs and HWs and shaped BF beliefs, experiences, and support.

“*When I gave birth, I didn't have enough breastmilk and the father didn't have money to supply food that can make the breast enough the children*.” Mother 8 [sic]

“*Sometimes you will go to an IDP camp, and you come across a mother and her children, eating some leaves, just only those leaves (…) You cannot say this mother is able to provide enough breastmilk for a baby.” Health promotor*

CGs sensed that emotional stress affected breastmilk production and hampered BF, and HWs perceived that stress negatively affected mothers' ability to breastfeed. Participants often verbalized how stress affected them as a result of conflict, displacement, and loss of livelihoods in the context of increasing poverty.

“*I think what makes my breastmilk not flowing is emotional stress because I think a lot… I am thinking how can I breastfeed this baby till 6 months, how can I get food for the rest of the family.” Mother 2*

“*This Boko Haram crisis caused a lot of poverty and displacement of people from their homes. So, people that initially, maybe they are not rich, but they used to farm and provide to eat. Now, because of the displacement they live no longer in their villages, they don't have anything.” Clinical staff*

From HWs' observations, breastmilk insufficiency was sometimes caused by inadequate BF practices in terms of positioning, latching, or frequency, even among multipara mothers. Mothers recognized their lack of knowledge of appropriate practices but did not identify this as a factor leading to breastmilk insufficiency.

“*Some do have breastmilk, but the method and the way they position, or the way they latch the baby or the times they take to breastfeed are normally the issues.” Clinical staff*

Another issue affecting BF was maternal medical conditions, including those affecting the breasts or the nipples. In some cases, mothers were discouraged from BF, for instance, when known to be HIV positive or presenting mastitis. When mothers had experienced previous neonatal or infant mortality, the death of the child is usually attributed to “poisonous” breastmilk, and normally mothers refused or were pushed to give up BF for further children. Most of the mothers interviewed were grand multipara. Some mothers felt their ability to BF changed over time, decreasing with older age and higher parity, since they were able to breastfeed their first children but experienced difficulties with later ones.

“*They believe if they give their child the breastmilk, the child may end up dying. Because already they are told that the breastmilk is bad. We use to face this challenge. And it is very difficult to convince the mother that the milk has no problem.” Health promotor*

### 3.3. Contribution of BF promotion to knowledge and practice

There was a shared perception that infant feeding practices were slowly improving over time. This change was due to the exposure of mothers to encouragement about exclusive BF through the radio, during antenatal care, promotion by NGOs, or after hospital delivery. It was perceived that communities were generally open to BF promotion messaging, and some families were willing to follow recommended practices.

“*Here in our community before we did not get the knowledge of breastfeeding babies only with breastmilk, so we breastfeed, we give water and also “pap” after 2 or 3 months. But now we have the knowledge of exclusive breastfeeding.” Grandfather*

“*They really understand and like the messages. Because there are some mothers that when they gave birth, I advised them about exclusive breastfeeding. And those mothers followed what I educate them, and they successfully breastfed their children until 6 months without giving water. And later they are appreciating… they never fed a child like this” Health promotor*

HWs identified several mechanisms contributing to the uptake of recommended feeding practices. One was the inclusion of key community members and traditional healers, as well as an open invitation to health promotion talks, including elderly family members. Another was the identification of cases that serve as an example of the positive result of promoted practices, following the idea that, “*seeing is believing”*.

“*Our people, they mostly believe in their traditional things. So that's why we used to include traditional healers and traditional birth attendants in our programs, so that we get more trust from the community.” Health promotor*.

“*I have a granddaughter who is living with me now, she has been fed only with breastmilk for the first 6 months. And from my observations, her health is different [better] from the other children who are fed with water, “pap” and breastmilk.” Grandfather (encouraging exclusive BF within his community)*

The **fear** of infants getting infectious diseases or developing malnutrition also guided the change in feeding practice, as BF promotion messages emphasized the risks of infants who were not breastfed.

“*They [at health facilities] are showing us pictures of malnourished babies as a result of giving formula and water for babies less than six… this led them to malnutrition and some other diseases (…) Everybody is afraid of disease, so that's why we decide to follow exclusive breastfeeding.” Mother 11*

Although mothers may have accepted the recommended practices, they often lacked the practical skills, self-confidence, or support to adhere to them. In those situations, the perceptions of breastmilk insufficiency prevailed over general BF promotion messages.

“*Giving the breastmilk only is the best way but most of the women are not able to produce enough breastmilk for the baby, so that's why we decided to introduce formula.” Mother 3* [sic]

“*The best way to feed an under 6 months old baby is exclusive breastfeeding, but if the breast is not enough for the baby, I will introduce other milk for the baby, because the baby will be malnourished if the breastmilk is not enough.” Mother 10*

The general strategy for health promotion within this MSF project included messaging on BF but it was not the main focus. HWs believed that general health messages did not always target the specific issues and multifactorial causes of lactation failure. HWs proposed several solutions to integrate BF support during health promotion activities more effectively. Proposed solutions included increasing the emphasis on BF physiology and practical skills, tailored messaging, and additional training for health promotors to enable them to provide this type of information and support.

“*If we send general messages, I don't think we will be able to make the connection with the women. (…) I am not convinced that they receive the messages that they need to know how to increase the milk production. Because I am not sure how much is focused on the timelines, how often they should feed…” Clinical staff* [sic]

### 3.4. Inpatient BF support as part of the management of malnutrition

#### 3.4.1. Challenges

CGs with infants <6 m normally came to the MSF nutrition project to seek care for malnourished or sick infants. On admission, most reported suboptimal feeding practices and complained of insufficient breast milk. However, their **expectation** was not to re-establish exclusive BF but to receive medical treatment for the infant, obtain free infant formula, or get a short medical treatment that could improve their breastmilk production.

“*They don't get the full picture that there is no medical intervention. It's just to eat well, drink a lot of fluids, then keep breastfeeding, and then child will improve slowly (…) The point is to help them produce milk, so that we can discharge on breastmilk only. But maybe they don't understand the whole idea of double suckling to increase stimulation and all that.” Clinical staff* [sic]

Before being admitted, CGs received explanations about the treatment, and they were also informed about a possible long length of stay. While feeling a bit overwhelmed by this change from initial expectations, staff clarifications, and reassurance helped them to adhere to treatment. Although CGs generally expressed their wish to stay admitted as long as needed to re-establish exclusive BF, they also raised concerns associated with a long stay. HWs pointed out that the duration of stay was a major problem in the care of infants <6 m. The willingness to stay decreased over time; as the health status of the infant improved, the CGs saw no reason to stay.

“*The issue is I left my children with my neighbor and my husband has travelled (…). I would like my baby to feel better early so I am discharged and go home to take care of my own children.” Mother 1*

“*Once the child is still on medication, they are normally ok, there is a treatment going, so they are ok. But once the medication finishes, and then they are still staying, the child is not sick, he is not having anything, they feel like. ‘why are we still here?”'. Clinical staff* [sic]

The mothers interviewed for this study were supported by their families, who encouraged them to stay and comply with the treatment at the ITFC. While many of their husbands were absent, they agreed for them to stay. However, HWs reported that this was not always the case. CGs sometimes felt distressed or powerless when admitted since they had left their family back home. Moreover, many CGs were emotionally unstable on arrival to the facility, given the vulnerability of the post-partum period, and the worries associated with infant feeding difficulties, sickness, or malnutrition.

“*To think about home is something we can't stop it, but they [MSF staff] always advise us to reduce it, because if it becomes much, it will affect us and the baby.” Mother 7* [sic]

HWs perceived a certain reluctance from women to accept direct BF support from male staff, due to a lack of trust in talking about “female issues”. CGs did accept care from male staff, although it reflected the differences and barriers between men and women in this culture.

“*The male and the female are different, so women feel more comfortable with the female staff than the male, and the male also sometimes doesn't feel comfortable because caregiver is a female, that's why there are feeling shy to come very close.” Mother 7* [sic]

“*They won't say they disagree, but then you know, facial expression matters a lot. Some don't relax, they don't tend to feel comfortable when is a male trying to assist them. While others don't care whoever it is.” Clinical staff (male)*

Acknowledging the numerous ethnic groups in the area, **cultural differences** between CGs and HWs—including the language barrier—also shaped the provision of BF support. When facing communication difficulties, HWs normally identified staff at the facility who could translate. Sometimes HWs felt frustrated and found it difficult to understand CGs' behaviors, practices, and decisions; this was a barrier to effectively engage with them.

“*She would tell you that she is so worried, but yet she doesn't want to breastfeed. If you are too concerned about your child's illness, or his condition, at least breastfeed him, but she won't.” Clinical staff*

While admitted, CGs demonstrated incorrect BF practices that sometimes the staff were not able to recognize (signs of ineffective suckling or BF only from one breast) or did not address for some time. From their viewpoint, this was due to a lack of time and knowledge. HWs reflected on possible solutions to improve staff capacities and practical BF support. They included the provision of practical, bedside training on different techniques. There was little engagement of nutritional assistants in direct BF support, so this could be addressed by training them for this purpose. Tools to assess BF practice and records of the progress could be better used to ensure a more systematic approach to BF support.

#### 3.4.2. Acceptance and gratitude

Despite initial expectations that were not met and the challenges in inpatient BF support, CGs gradually adapted to the routine at the facility and most accepted and complied with the recommendations to increase breastmilk production. The uptake of supplementary suckling technique was good overall and CGs and infants slowly demonstrated improved BF practice while hospitalized.

“*It's strange for me to give the breast together with the tube for the baby, but I am not finding difficulties to do it.” Grandmother 1*

“*I think it really helped my baby because if I would use a cup for my baby, I think half of the milk will pour down. But by using the tube, the baby will take almost all the milk.” Mother 7*

Different enablers contributed to this acceptance. The **role of mental health counselors seemed crucial** for BF support in this context. Their support was constantly required by other clinical staff, as they—as well as some health promotors—were the ones who were able to effectively encourage GCs to follow advice. They followed a culturally sensitive approach and supported women emotionally by listening to them, talking to them calmly, and encouraging them with positive examples.

“*Maybe sometimes the doctor wants to do some important, but because of the refusal they have to stop (…) But if there's maybe a HP or counsellor to help them, they are very comfortable… What can you do? You cannot deny culture” Clinical staff*

CGs also supported each other and encouraged other CGs to follow recommendations, after seeing the improvements with their infants.

“*Caregivers also encourage themselves. Even when they are depressed and one says 'I don't have breastmilk', they use themselves as examples, to tell them 'When I came in, I didn't have milk, but due to the counseling, and the treatment doctors, nutrition assistants, the nurses, everybody… the support, and then the milk comes out.” Clinical staff*

At the same time, BF support was included in the overall comprehensive treatment provided—rest, appropriate nutrition, and care for the infants—and mothers felt well treated and comfortable at the facility; this is an enabling environment to breastfeed. Above all, CGs saw improvements in infants' health and nutritional status, and also for themselves. All interviewed mothers felt grateful for the services provided and were willing to recommend the uptake of BF to other CGs.

“*The women feel comfortable being at the ward and we use to sit down and have a chat, breastfeed our children… sometimes we are even with the staff” Mother 7*

“*When I was admitted I received a lot of support and guide from the staff. I feel comfortable with them and… there are meals, there are food, everything in the facility is ok. When I went back home my family were happy to see how I have changed.” Grandmother 3* [sic]

#### 3.4.3. Dilemmas of infant formula demand and supply

On admission, CGs are told that accepting BF support is part of the treatment process during hospitalization and that infant formula is not provided upon the mother's desire. HWs highlighted a lack of a straightforward solution for mothers not wishing to breastfeed. Yet, they thought that a more flexible approach to formula provision will cause an increase in formula feeding demand and consumption—and a subsequent increase in risks of artificial feeding. Infant formula in this context is an expensive product and HWs reported how some CGs obtained it for free then sold it to get money; this also happens with other therapeutic supplements given at the facility. HWs described a few scenarios that frequently lead to formula supply after BF support, such as wet nursing or multiple births. They perceived that there was a lack of unified criteria for formula provision in some cases, for example, when mothers were not achieving exclusive BF after a period of BF support.

“*We know all the advantages of breastfeeding… we know that it's free, we know that it's hygienic. So I would rather improve our services [BF support] than to make infant formula a convenient option (…) I think there should be a clearly defined criteria when to switch to formula” Clinical staff* [sic]

### 3.5. Follow-up and community-based support

When discharged, infants were followed up weekly at ATFC until they recovered. Some CGs and HWs feared that BF practices would decrease or stop after discharge since CGs lacked access to appropriate food, staff encouragement, and support at home. Both CGs and HWs strongly believed that maternal nutrition was crucial for an adequate supply of breastmilk, something that was not often possible at home.

“*For the purpose of their care in the facility, they will breastfeed, because we encourage them, and we do psycho-education… so they do it. But when they are being discharged home, and when they come up for follow-up, we find that the child has already stopped.” Clinical staff* [sic]

“*When I was in admission in the facility, they gave us food (…) so it helped the breast to start producing breastmilk. But immediately when I went back home because I can't afford to buy this kind of food and eat, the supply of the milk is going down.” Grandmother 3 (attending for follow-up at ATFC)* [sic]

It was perceived that ATFC services, traditionally targeting only older children, were not including BF promotion or support during follow-up visits but were focused on following up on weight changes or general health status. HWs suggested that BF promotion and support should be included in a stronger community-based approach to ensure sustainability after the hospital interventions.

“*When they come, we treat and then they go back, but there is still a gap, so it should be community-based (…) This is where we should start these cultural beliefs… If we don't do it, no matter what you do, they'll come back.” Clinical staff* [sic]

## 4. Discussion

The experiences and perspectives shared by participants portray a setting in which BF is practiced by most mothers, but the uptake of exclusive BF seems low. In a survey exploring infant feeding practices in Borno State, only 33% of caregivers reported having fed their children only with breastmilk for the first 6 months of life (37). Available reports suggest an increase in exclusive BF rates over time, as perceived by participants in this study (38–40).

This study exposes enabling and disabling factors for BF practice and support perceived by CGs and HWs in the studied context, which are summarized in Table 3. Yet, many of these perceived factors have also been identified in other humanitarian and low-middle-income settings and among mothers and infants globally (18, 41). While medical humanitarian organizations may not directly tackle structural barriers—such as household dynamics, traditional beliefs, displacement, and food insecurity—these factors should be recognized and addressed when developing strategies to promote and support BF. At the program level, BF promotion messages and support activities should be adapted to the common barriers to BF reported in this specific context. There is a need to strengthen HWs' capacities for lactation support while providing them with knowledge and skills to effectively counsel women so they can gain confidence in their ability to breastfeed despite being stressed or having poor nutrition. Staff training should also be enlarged to a wider range of healthcare roles, such as nutritional assistants or health promotors. Specific areas for improvement are mentioned throughout this discussion.

**Table 3:** Summary of perceived enabling and disabling factors for BF practice, promotion, and support.

**Enabling factors**	**Disabling factors**
**Individual and community level**	
- Mothers' wish to breastfeed, BF culturally considered a maternal duty - Family support and encouragement - Acceptability of wet nursing by grandmothers - Motivation to re-establish BF when understood as part of the treatment for malnourished infants - BF promotion at different levels (media, antenatal care, NGOs)	- Maternal death or medical conditions, multiple births - Lack of BF skills - Certain traditional beliefs, discouraging messages from traditional healers - Unsupportive family members - Hot and dry weather conditions - Frequent perception of breastmilk insufficiency due to food insecurity and stress - Lack of initiative to seek skilled BF support when BF difficulties encountered
**Program level**	
- BF promotion inclusive of family members, community leaders and traditional medicine providers - Identification of positive examples (healthy infants exclusively breastfed) - Comprehensive inpatient care, including appropriate nutrition, rest, and skilled BF support - Frequent involvement of mental health counselors - Peer encouragement and support	- Generic health promotion messaging not addressing specific BF difficulties/barriers - Limited capacities for lactation support by health promotors or community health educators - CGs expectation to receive formula milk from humanitarian organizations - Cultural or gender differences between CGs and HWs - HWs overemphasis on the need for appropriate nutrition among lactating mothers - Reluctance for a long length of hospital stay - Outpatient and community support poorly adapted for infants < 6m

Interviewed mothers were generally disempowered by infant feeding decisions or healthcare-seeking behaviors. This has been already shown in other studies looking at social norms in Borno state (42) or focusing on African cultures (43). This limitation of mothers' autonomy should be considered when providing BF messages and support, by involving key family members, when possible. In this setting, we found grandmothers who were willing to wet nurse accepted to be hospitalized with their grandchildren to receive inpatient BF support as part of the treatment for infant malnutrition. However, there was a perception that wet nurses seldom achieved exclusive BF despite support. Future studies should assess the outcomes of BF support among wet nurses, including infant nutritional recovery. Similarly, there is a need to increase staff capacities and explore strategies to provide enhanced support for wet nurses, acknowledging the high maternal mortality ratio observed in Nigeria and other humanitarian settings (44).

The conflict and displacement in Maiduguri have augmented the risk of acute malnutrition among infants and reduced dietary availability for mothers (2). Food insecurity can impact BF practice through various pathways. It can compromise maternal nutrition, not only affecting mothers' wellbeing but also leading to the perception of insufficient breastmilk and resulting in mixed feeding or premature cessation of BF. Additionally, food insecurity can induce stress or depression, often triggered by the inability to provide enough food for the family (45–49). Stress does not directly affect breast milk production. However, it can temporarily delay breastmilk release through reduced oxytocin secretion and reduced maternal self-confidence, as well as impact mother–infant behaviors (e.g., maternal anxious behavior subsequently increasing infants' irritability during feeds) (50–52). These mechanisms potentially affecting BF practice could be overcome when providing holistic support and reassurance. Offering mental and psychosocial support for lactating mothers is particularly relevant to promoting maternal wellbeing and protecting and enabling BF in humanitarian settings, where stressors likely increase (20, 53).

Generally, CGs thought reduced breastmilk production and flow were irreversible and felt powerless to change that situation. To reverse this perception, sensitive counseling can provide information and support to reinforce maternal self-efficacy and lead to improvements in BF (18). Messages should focus on the mechanisms of breastmilk production and how it can be increased (49). CGs and HWs should improve their understanding and management of certain infant behaviors, such as fussiness or crying, that often led to direct feeding supplementation and discontinuation of BF (54).

In this context, HWs strongly emphasize the importance to increase the intake of fluids and nutritious food for lactating mothers. It has been previously documented how these messages can undermine BF practice in contexts where mothers cannot reach the recommended intake (53). Up to now, there is not enough evidence suggesting the need to increase fluid intake beyond what lactating women require to meet their physiological needs (55). Breastmilk production and macronutrient composition seem to be little affected by maternal intake although maternal micronutrient deficiencies appear more reflected in breastmilk. In the case of severely malnourished mothers, BF can be extra challenged by energy depletion and the mother feeling unwell. While mothers can maintain lactation even when having a poor diet and nutritional status, more research is needed to investigate the extent of the influence of these factors on infant growth (56–59). Nonetheless, in resource-limited settings, exclusive BF likely remains the most complete and safe option for infants <6 m and should be strongly promoted and supported (60). The messages encouraging a healthy diet among lactating mothers should be delivered sensitively, in coherence with mothers' access to food, and coupled with maternal nutritional support when possible. At the same time, maternal nutritional status should be assessed and considered to better support mothers and infants.

The Operational Guidance for Infant Feeding in Emergencies outlines the limited circumstances when infant formula should be provided in emergencies (6). These recommendations arise with the purpose to protect mothers and infants from a wide distribution of formula, which may discourage mothers from re-establishing BF and undermine overall BF practice in the community (61, 62). However, as previously documented, the guidance is sometimes not understood or difficult to follow by those translating recommendations into practice. For instance, HWs may feel they are dismissing maternal autonomy or may hesitate to decide when BF support is considered ineffective and infant formula should be provided in those cases (8, 14). Staff training sessions should encourage discussions around the practical application of guidelines in a specific context, acknowledging HWs' dilemmas and experiences. Further research should also focus on identifying the best ways to support non-breastfed infants in emergencies.

Within this project, inpatient BF support worked effectively to help achieve partial and exclusive BF. CGs generally accepted the use of supplementary suckling techniques and the advice to stimulate BF (e.g., frequent suckling and bedding-in). The acceptability and outcomes of relactation support can vary widely among contexts (46, 63). The success in the studied setting was likely linked to peer support and the constant encouragement by HWs in a clinical setting where the use of supplementary suckling was well established. One of the main issues challenging BF support in the facility was the prolonged length of stay in some cases. This was also mentioned by CGs receiving relactation support in Kenya (46). As proposed by HWs, the feasibility and outcomes of outpatient support or intermittent admission—allowing mothers to go home for a few days and return to continue BF support—could be considered in this context. This study highlighted several reasons to consider a community-based approach for the management of malnourished infants <6 m. The Community Management of At-risk Mothers and Infants under 6 months of age (C-MAMI) care pathway, already supports and guides this approach, although its practical applicability and impact are being evaluated (64).

Several study limitations should be considered. Participant CGs had already accepted treatment at the facility. Hence, CGs who refused MSF treatment, who may have had different views, were not included in the sample. We recruited participants reachable at the facility or IDP camp during the limited data collection period, which did not allow interviewing other GCs (e.g., father and adolescent mother). A degree of social desirability bias must be considered in the answers recorded. HWs may have had a sense of performance evaluation that might have changed their opinions. CGs were receiving free care from MSF and were also offered an incentive for their participation in the study. This bias was mitigated by triangulating information from different sources, researchers' observations, and discussions among the research team in the field (65). Finally, the results of this study captured findings in a particular setting and time, so that they may not be generalizable to other contexts or more acute emergencies.

Since this operational research study aimed to create evidence-based practice, the authors disseminated the results among MSF field staff and managers in the Maiduguri project. It was hoped that this information would contribute to improving infant feeding support in this specific context (Supplementary material 2). These operational recommendations could be applied in other similar humanitarian contexts.

## 5. Conclusion

This study re-emphasizes that the practice of BF does not only depends on mothers' wishes, knowledge, and skills but it is strongly influenced by household and contextual factors. The perception of breastmilk insufficiency linked to poor maternal nutrition is one of the most common barriers to BF in this setting and should be particularly addressed during BF promotion and support. Within the studied project, BF support seems to contribute to improvements in BF practice, and it is positively valued by CGs, despite challenges and areas for improvement in HWs' capacities. A key factor enabling the acceptance of BF support was its integration as part of the comprehensive care provided to CGs and malnourished infants during admission. A greater focus should be placed on providing targeted support and follow-up for infants <6 m and their CGs in the community.

## Data availability statement

Data could be available upon reasonable request and with permission from Medecins Sans Frontiers. Requests to access the datasets should be directed to nieves.amat.camacho@ki.se.

## Ethics statement

The studies involving human participants were reviewed and approved by MSF Ethics Review Board (ID 2207) and the Borno State Health Research Ethics Committee, Nigeria (Approval No. 113/2022). The patients/participants provided their written informed consent to participate in this study.

## Author contributions

NAC, EB, AC, TS, JVS, OK, and UP contributed to study design. HK, FH, AC, and CB supported data collection process. NAC collected and analyzed the data with contributions from AC, EB, UP, and TS. NAC drafted the first version of the article. All authors contributed to the discussion of results and read and approved the manuscript.
