# Supplementary material for: Promoting and supporting breastfeeding in a protracted emergency setting—Caregivers' and health workers' perceptions from North-East Nigeria
Source: Front Public Health. 2023 Jun 2;11:1077068. doi: 10.3389/fpubh.2023.1077068 (PMC10272820; doi:10.3389/fpubh.2023.1077068)
Supplement: Supplementary file 1 [file Table_1.DOCX]

Supplementary Material 1

# In-depth Interview Guide

Before conducting the interview:

- Interviewer and translator will introduce themselves
- Explain the objective of the IDI and thank the interviewee for his/her participation
- Explain what it will happen during the interview and how long will it take. Remind that they can stop the interview at any time.
- Remind that there is no right or wrong answers and their responses will not affect negatively any care they receive afterwards.
- Verification of informed consent and permission for recording.
- Allow any questions from participant.

**For mothers/caregivers**

1. **Infant feeding practices**

- Please tell us about your experience feeding your infant
- Would you tell us about the way the baby is normally fed (or was fed when aged less than 6 months)?

*Probes:*

- Who does feed the baby?
- How many times a day do you feed him/her?
- (If not EBF) What liquids/foods do you use (e.g. artificial milk, other food,...)? Why? How do you get those? Is it difficult to get them (e.g. buy, donation...)?
- (If not EBF) What do you use to feed the baby (e.g. bottles, cups...)? How do you maintain them clean?

1. **Barriers and enablers to BF**

- How do you decide what and how to feed the baby?
- Ideally, what do you think is the best way to feed babies until 6 months of age?
- What helps you to breastfeed/exclusively breastfeed the baby?
- What makes it difficult to breastfeed/ exclusively breastfeed the baby?

*Probes*:

- What do you think are the benefits to breastfeed your baby?
- What do you think are the downsides of breastfeeding your baby? (did you face difficulties while breastfeeding?)
- Were you planning to breastfeed before the baby was born?
- Did you receive support/felt supported for BF (e.g. teaching you how to do it, how to solve problems...)? What kind and from whom did you receive support?
- How did you learn about infant feeding practices?
- What is the common practice for feeding small babies in the community? Is it recommended to only breastfeed until babies are 6 months old?
- How is breastfeeding seen/perceived in your culture? Why do you think is that?
- Are you happy/comfortable to breastfeed (for mothers)?
- Does your family, or anyone else, advise and help you to breastfeed? How?
- Have mother or baby had any health problems that make them not to breastfeed?
- Has the conflict/displacement in the area affected the way the baby is fed? How?
- Do you think what you eat affects breastfeeding (e.g. if you are not having enough food)?
- How does the care of other children, or other family events, influence in your decision to breastfeed?
- How does work and domestic tasks influence in your decision to breastfeed? Was breastfeeding (or not) an additional burden?

1. **Experiences receiving BF support**

- What sources do you go to for information about feeding your baby?
- Why do you trust those sources?
- What information about BF have you received from MSF?
- What help have you received from MSF to re-establish or improve BF?
- How did you feel about receiving this type of information or support?
- How did you feel about the breastfeeding support? Did it have any affect on your daily life, or on the way you feed your baby?
- Did you find it was possible for you to follow health workers recommendations?
- How did you feel interacting with health workers supporting you to breastfeed?

1. **Preferred or required support for BF**

- What could help you (or have helped you) to breastfeed/ exclusively breastfeed the baby?
- Who would you like to play a role in helping you to breastfeed the baby?
- How could MSF better help you with breastfeeding or artificially feeding your baby?

*Probes*:

- Who do you think can help you to breastfeed?
- How can you get help with breastfeeding?
- Have you ever given or received help with breastfeeding? Can you give an example?
- Do you think health workers can help you to breastfeed?
- (If relevant) would you be interested in receiving support to re-establish BF (relactation)?
- (If relevant) would you consider the baby to be breastfed by another woman (wet nursing)? How is that option considered in your community/culture?
- (If BF is not an option) What would you need to feed the baby in the best possible way if not breastfed?

**For family members**

- What is the most common way to feed babies in your family and community nowadays?
- Have feeding/BF practices changed overtime? How? Reasons: Conflict, displacement, access to formula?
- Ideally, what do you think is the best way to feed a baby until 6 months of age?
- Do you participate in the feeding of the baby? Do you support the mother?
- Have you given the mother advise or support? Which type of information or support?
- What are the difficulties that mothers/caregivers find to feed their babies?
- What support do you think the mother/caregiver need to feed the baby in the best way?
- Are you aware of the information and support MSF provides to help BF?
- What do you think about it?
- Do you trust MSF staff can help your family to feed the baby in a better way?

**Focus Group Discussion (FGD) Script**

- FGD leader (PI) will introduce herself
- Introduction of participants
- Explain the objective of the FGD and thank participants for their involvement
- Explain what it will happen during the session, ground rules and how long will it take. Remind they can stop at any time.
- Remind there is no right or wrong answers and their responses will not affect their employment status at all.
- Verification of informed consent and permission for recording.
- Allow any questions from participant.

Questions and desirable topics for discussion

The PI will bring up the following questions and only mention the proposed probes underneath if those are not mentioned by the participants and seem relevant during the discussions.

**FGDs with MSF staff**

1. **What do you perceive are the most common feeding practices for infants <6m in this setting?**

- Most prevalent feeding practice in the community?
- How is BF perceived among women in this context?
- Frequency, access and use of formula feeding? Other common artificial feeding practices?

1. **Which** **barriers to BF do women report or you perceive they have in this context?**

- What is the general knowledge on how to BF and solve rising problems?
- Health problems of the mother or infant?
- Baseline practice: is BF common, formula use?
- Community: beliefs, cultural or religion conceptions
- Family support or discouragement? Number of children to care for
- Conflict: affected by displacement?
- Living conditions, poverty

1. **How is BF promoted and supported within the project?**

- Who is the staff involved?
- How is relevant information provided?
- How are mothers practically supported to BF in different situations? For example, if not BF or if partially BF
- How do you perceive mothers react to BF promotion and support in this context?

1. **Which** **barriers do you find to promote and support BF in this context?**

- Health workers beliefs and motivation about BF support. Do you feel prepared to promote BF in this context? How do your own experience, knowledge, beliefs influence this?
- Cultural differences between health workers and mothers targeted for BF support
- Mothers’ perceptions about the way BF is promoted and supported
- Project related: dedicated time and space? appropriate knowledge and skills of health workers? protocols, available guidelines? work overload, compatibility with other tasks?
- Community vs MSF approach to provision of breastmilk substitutes (BMS) (i.e. formula)?
- Influence from BF promotion or BMS supply by other actors?

1. **How do you think BF promotion and support could be improved in this setting**

- What actions have helped or could help overcoming previously mentioned barriers
- Can you mention concrete experiences when BF support worked to increase BF practice?
- Examples when BF support failed, or when it was needed but not provided?
- What do you think will help mothers to BF in this context at different levels? MSF project, community, other local/international actors
