# Supplementary material for: Promoting and supporting breastfeeding in a protracted emergency setting—Caregivers' and health workers' perceptions from North-East Nigeria
Source: Front Public Health. 2023 Jun 2;11:1077068. doi: 10.3389/fpubh.2023.1077068 (PMC10272820; doi:10.3389/fpubh.2023.1077068)
Supplement: Supplementary file 2 [file Table_2.DOCX]

Supplementary Material 2

**Operational considerations for practice**

- The influence of the closest family members (commonly the husband and the grandparents) on infant feeding and childcare practices should be always considered while providing messages and support. The advice by traditional healers, spiritualists may be contradictory to the one providing by MSF, and this should be also considered. The inclusion of traditional healers or traditional birth attendants is an important entry point in the community, and a possible way to expand health promotion (HP) messages.
- HP strategies not only focus on BF among young infants, but on several nutrition and public health components. Therefore, information regarding exclusive BF may be diluted and fail to provide specific messages for mothers with infants <6m encountering BF difficulties. General information will less likely reach and help mothers with specific problems. Health promotors could dedicate some time to identify infants <6m and assess and target their specific needs. The feasibility of the approach proposed by the C-MAMI care pathway (to assess, identify, and treat malnourished infants <6m and their mothers in the community) could be studied.
- Regarding HP messaging, more emphasis should be placed on reinforcing the reversibility of breastmilk insufficiency and the mechanisms to increase breastmilk production. It is common that mothers know that exclusive BF is the best for their babies, but they feel in their own case this is not possible to accomplish. It could be useful to provide information about the possible cause of certain infant behaviors (not always reflecting that the baby is hungry) and give advice on how to manage those, following locally accepted practices. This may prevent some of the cases of quick start of fluid or food supplementation.
- When talking about the importance of maternal nutrition for breastmilk production, HWs should be aware of the food availability for this mother-infant household or community and how these messages may be interpreted. A sensitive and coherent narrative should be agreed upon among HWs in order to prevent a reduction in mothers BF self-efficacy. Parallelly, efforts to ensure the appropriate nutrition of the mother should be made, for instance, by establishing referral systems with other organizations if possible.
- The identification and training of community peer-supporters (mothers who can help other mothers to breastfeed) can provide an easier access to support for women in the community, which may be better accepted, and increase inpatient support capacity especially during the hunger period or other overwhelming emergency peaks.
- Clinical staff could increase the use of different techniques to increase breast stimulation, such as breast massage, manual expression of the breast or the use of the breast pump not only to measure the quantity of milk produced. Practical training could be valuable, targeting nurses, midwives, nutritional assistants, and if possible identified health promotors, who can all support mothers during feeding times, and in that way increase capacity. A more systematic assessment of BF practice could benefit BF support and will prevent HWs for missing important aspects to assess and manage. Available tools can be applied.
- The household characteristics, marital relationship, and other support (or lack of) at home should be understood when asking women to stay in the facility for long periods (e.g., how other children will be cared for back home). Consider alternatives to reduce or ease long length of stay at the ITFC, for example, the feasibility of intermittent admission or the provision of community-based support, as above mentioned.
- The criteria applied for the provision of infant formula can be interpreted differently among the clinical staff assessing each case, and the context in which decisions are taking can change overtime (e.g., worsening situation leading to an acute emergency phase with less resources available). Those decisions sometimes entail a moral dilemma for the staff. Multidisciplinary team training should be provided on the practical use of guidelines in this specific context, and debriefing sessions should be organized to discuss doubts, dilemmas, or operational difficulties expressed.
- The follow up visits of infants <6m at ATFC should be better structured to meet the needs of infants in this age group. Visits should be an opportunity to monitor feeding practice, identify possible difficulties or problems and provide (or refer for) BF support if needed.
- The collection of routine data in the facility could be better adapted for infants <6m. For example, maternal variables such as age, or nutritional status (e.g., using MUAC/BMI) should be recorded, as well as the specific infant feeding practices on inpatient admission and discharge, and each of the outpatient visits.
